# *Plasmodium* Circumsporozoite Protein Enhances the Efficacy of Gefitinib in Lung Adenocarcinoma Cells by Inhibiting Autophagy *via* Proteasomal Degradation of LC3B

**DOI:** 10.3389/fcell.2022.830046

**Published:** 2022-02-03

**Authors:** Xiao Lu, Jiao Zhang, Yan-Qi Li, Quan-Xing Liu, Dong Zhou, Xu-Feng Deng, Yuan Qiu, Qian Chen, Man-Yuan Li, Xiao-Qing Liu, Ji-Gang Dai, Hong Zheng

**Affiliations:** ^1^ Department of Thoracic Surgery, Xinqiao Hospital, Army Medical University, Chongqing, China; ^2^ Department of General Surgery, Xinqiao Hospital, Army Medical University, Chongqing, China; ^3^ Cancer Center of Daping Hospital, Army Medical University, Chongqing, China

**Keywords:** lung adenocarcinoma, circumsporozoite protein, gefitinib, autophagy, ubiquitination

## Abstract

**Background:** Almost all lung adenocarcinoma (LUAD) patients with EGFR mutant will develop resistance to EGFR-TKIs, which limit the long-term clinical application of these agents. Accumulating evidence shows one of the main reasons for resistance to EGFR-TKIs is induction of autophagy in tumor cells. Our previous study found that circumsporozoite protein (CSP) in *Plasmodium* can suppress autophagy in host hepatocytes. However, it is unknown whether CSP-mediated inhibition of autophagy could improve the anti-tumor effect of EGFR-TKIs.

**Methods:** We constructed A549 and H1975 cell lines with stable overexpression of CSP (OE-CSP cells). CCK-8, Lactate Dehydrogenase (LDH), flow cytometry, and colony analysis were performed to observe the effect of CSP overexpression on cell viability, apoptosis rate, and colony formation ratio. The sensitizing effect of CSP on gefitinib was evaluated *in vivo* using a subcutaneous tumor model in nude mice and immunohistochemical assay. The role of CSP in regulation of autophagy was investigated by laser confocal microscopy assay and western blotting. A transcriptome sequencing assay and real-time polymerase chain reaction were used to determine the levels of mRNA for autophagy-related proteins. Cycloheximide (CHX), MG132, TAK-243, and immunoprecipitation assays were used to detect and confirm proteasomal degradation of LC3B.

**Results:** OE-CSP A549 and H1975 cells were more sensitive to gefitinib, demonstrating significant amounts of apoptosis and decreased viability. In the OE-CSP group, autophagy was significantly inhibited, and there was a decrease in LC3B protein after exposure to gefitinib. Cell viability and colony formed ability were recovered when OE-CSP cells were exposed to rapamycin. In nude mice with xenografts of LUAD cells, inhibition of autophagy by CSP resulted in suppression of cell growth, and more marked apoptosis during exposure to gefitinib. CSP promoted ubiquitin-proteasome degradation of LC3B, leading to inhibition of autophagy in LUAD cells after treatment with gefitinib. When LUAD cells were treated with ubiquitin activating enzyme inhibitor TAK-243, cell viability, apoptosis, and growth were comparable between the OE-CSP group and a control group both *in vivo* and *in vitro*.

**Conclusion:** CSP can inhibit gefitinib-induced autophagy via proteasomal degradation of LC3B, which suggests that CSP could be used as an autophagy inhibitor to sensitize EGFR-TKIs.

## Introduction

Lung cancer is a common malignancy with high morbidity and mortality rates and results in 1.8 million deaths each year (Bray et al., 2018). Non-small cell lung cancer (NSCLC) accounts for 85% of all lung cancers, and lung adenocarcinoma (LUAD) is the most common subtype. Although a conventional therapeutic strategy based on surgical resection followed by chemoradiotherapy can benefit patients with early-stage LUAD, most patients have advanced LUAD at the time of diagnosis and a poor prognosis after conventional treatment (Howlader et al., 2020). Epidermal growth factor receptor (EGFR)-tyrosine kinase inhibitor (TKI) therapy is preferred in patients with locally advanced or metastatic NSCLC because of its high specificity, curative effect, and favorable safety profile. Although activating EGFR mutations are only found in 10–15% of LUAD in white populations and in 40–50% of cases in Asian, EGFR-TKIs have doubled the disease-free survival rate in patients with advanced NSCLC and an EGFR mutation (Maemondo et al., 2010; Arbour and Riely, 2019; Westover et al., 2018). Unfortunately, drug resistance is a crucial issue in the clinical application of EGFR-TKIs. The majority of LUAD patients with EGFR mutant rapidly develop resistance during EGFR-TKI therapy and do not benefit from treatment (Ohashi et al., 2013). Many efforts have been made to overcome this resistance, including combining an EGFR-TKI with an anti-neovascularization agent (Passaro et al., 2021) or c-Met inhibitor (Friese-Hamim et al., 2017). Despite some positive overall results, these strategies cannot solve the resistance problem completely. Therefore, there is an urgent need to develop new targets to combat drug resistance.

Autophagy is a highly conserved survival process that is precisely regulated to degrade aging organelles or malfunctioning proteins in response to stress, hypoxia, and or starvation (Rubinsztein et al., 2012). Interestingly, autophagy has opposite functions under different conditions. In normal somatic cells, autophagy degrades misfolded proteins to prevent malignant transformation. However, in an established tumor, autophagy can act as a survival mechanism that prevents tumor cell death when exposed to harsh conditions and chemotherapy (Poillet-Perez et al., 2021; Rao et al., 2014; Wang et al., 2016). Moreover, autophagy is upregulated in response to treatment with an EGFR-TKIs and plays a critical role in resistance to TKIs (Fung et al., 2012; Li and Fan, 2010). Combined treatment with an EGFR-TKI and an autophagy inhibitor may be an effective approach when basal autophagy is upregulated in cancer cells (Sakuma et al., 2013; Su et al., 2016; Uberall et al., 2019). There is some evidence indicating that inhibition of autophagy is a potential target that could be used to overcome resistance to EGFR-TKIs (Mulcahy and Thorburn, 2020).

Cancer gene therapy is a promising method to treat cancer by correcting defective genes or transferring therapeutic nucleic acids into tumor cells (Hu et al., 2021). Circumsporozoite protein (CSP) is a surface protein of the *Plasmodium* sporozoite and has a critical role in the motility of the sporozoite and its ability to invade a host (Livingstone et al., 2021). After sporozoites invade a host hepatocyte, CSP is shed and released into the cytosol to modulate intracellular signaling pathways that assist in the development and survival of parasites (Zheng et al., 2014). Our previous study found that CSP could suppress autophagy in hepatocytes, thereby helping the parasite to evade interferon-gamma-mediated death (Zheng et al., 2021). This finding raised the interesting possibility that CSP overexpression might increase the sensitivity of LUAD cells to EGFR-TKIs. Therefore, the aim of the present study was to explore the role of CSP-mediated inhibition of autophagy in EGFR-TKI resistance in LUAD cells.

## Materials and Methods

### Cell Culture and Reagent

LUAD cells (A549 and H1975) and HEK293T were purchased from Cell Bank of Typical Culture Preservation Committee, Chinese Academy of Sciences, Shanghai (Shanghai, China), and A549 and H1975 cells were cultured in Dulbecco’s Modified Eagle Medium (Hyclone Laboratories, Logan, UT, United States) with 10% fetal bovine serum (Gibco Laboratories, Gaithersburg, MD, United States), 1% penicillin-streptomycin, and 2 mM L-glutamine (Sangon Biotech, Shanghai, China) at 37°C in a humidified atmosphere of 5% CO_2_. HEK293T cells were cultured in Roswell Park Memorial Institute (RPMI) 1,640 Medium (Hyclone) with supplements described above.

### Construction on Circumsporozoite Protein Stable Expression Lung Adenocarcinoma Cells

The *Plasmodium yoelii* CSP coding sequence was obtained in the manner described in our previous study (Ding et al., 2012) and subcloned into pLenti plasmid (pLenti-CSP). pLenti or pLenti-CSP plasmids were transfected into HEK293T cells with pMD2. G and psPAX2 plasmids to created lentiviruses for control and stable overexpression of CSP cells (OE-CSP cells). A549 and H1975 cells were infected with control or CSP-overexpressing lentiviruses at a multiplicity of infection of 15 for 48 h. Cells were limited-diluted and monoclonal-cultured during puromycin challenge (3 μg/ml, Gibco) to select infection-positive cells. The control and OE-CSP cells were cultured in Dulbecco’s Modified Eagle Medium supplemented with 10% fetal bovine serum and 1 μg/ml puromycin.

### Cell Viability Assay

Cells (2 × 10^3^) were seeded in 96-well plates and cultured for 12 h. For detection of viability, the cells were treated with various concentrations of gefitinib for 24 h or 25 µM gefitinib for the indicated time points. Cell viability was examined using a CCK8 kit (Beyotime Biotechnology, Jiangsu, China).

### Lactate Dehydrogenase Assay

Cellular cytotoxicity was detected using a Lactate Dehydrogenase (LDH) assay kit (Beyotime Technology) according to the manufacturer’s instructions. Briefly, cells (5 × 10^3^) were seeded in 96-well plates and treated with gefitinib for 24 h. After treatment with gefitinib, the LDH release rate was detected at 490 nm, and the percentage of LDH released was calculated for cellular cytotoxicity.

### Apoptosis Assay

Cells (5 × 10^5^) were seeded in 12-well plates and treated with 25 µM gefitinib, 0.5 μg/ml rapamycin, 1 µM TAK-243 (MedChemExpress, Monmouth Junction, NJ, United States), and or combined treatment for 24 h. Annexin V (BioLegend, San Diego, CA, United States) and propidium iodide (Beyotime Technology) were stained on the surface of the cells. The data were analyzed using FlowJo software (version 10.0; Ashland, OR, United States).

### Colony Formation Assay

Cells (1 × 10^3^) were seeded in 6-well plates and cultured for 12 h. The cells were then treated with 5 µM gefitinib for 7 days. The clones were fixed with 4% paraformaldehyde and stained with 0.1% crystal violet solution. The colony formation assay was repeated three times. All data were analyzed using ImageJ software (National Institutes of Health, Bethesda, MD, United States).

### Immunofluorescence Assay

Cells were seeded on the surface of 8-mm × 8-mm slides and cultured for 12 h. Cells were infected with the RFP/GFP-LC3B adenovirus after treatment with polybrene to neutralize the negative charge on the cell surface. The medium was replaced with Hank’s balanced salt solution (HBSS). Twenty-four hours later, the cells were harvested, fixed with 4% paraformaldehyde, and underwent nuclear staining with DAPI. DAKO Fluorescence Mounting Medium (Agilent Technologies Inc., Santa Clara, CA, United States) was used to avoid fluorescence quenching, and the slides were mounted for fluorescence microscopy. Autophagic flux was visualized and analyzed via fluorescence imaging of autophagosomes and autolysosomes as introduced in the previous publication (Gump and Thorburn, 2014; Klionsky et al., 2021; Mizushima and Yoshimori, 2007).

### *In vivo* Xenograft Model

Control cells or OE-CSP cells (5 × 10^6^) were mixed with Matrigel in a volume ratio of 1:1 and subcutaneously injected into nude mice (Charles River Laboratories, Beijing, China). When the tumor volume was approximately 100 mm^3^, the mice were divided into control-NC, CSP-NC, control-gefitinib, and CSP-gefitinib groups. The control-NC and CSP-NC groups were fed olive oil every other day, while the control gefitinib and CSP-gefitinib groups were treated with suspension buffer containing gefitinib (150 mg/kg) and olive oil. Mice were treated for 3 weeks with TAK-243 (Hyer et al., 2018) (intravenous administration at 2 doses/week, 12.5 mg/kg) or plus gefitinib to detect the role of the ubiquitin-proteasome system. The long 1) and short 2) tumor diameters were measured with a Vernier caliper, and the computational formula used for tumor volume was v = a*b^2^/2. Tumors were extracted, photographed, and fixed with 4% paraformaldehyde for immunohistochemical assay. Nude mice were purchased from the Vital River Laboratory Animal Technology (Beijing, China). Animals were kept in a specific pathogen-free laboratory at the Institute of Immunology of Army Medical University. All methods were carried out in accordance with the approved Guide for the Care and Use of Laboratory Animals of the Army Medical University.

### Immunohistochemical Assay

The immunohistochemical assay protocol was performed as described in a previous study (Zhang et al., 2021). Briefly, the tumor tissues were embedded in paraffin, sectioned, and dewaxed, after which the antigen was restored in citrate buffer. The sections were incubated with TUNEL, Ki67 (Thermofisher, United States), and LC3B (anti-LC3B, Cell Signaling Technology, United States) to detect cell apoptosis, proliferation, and autophagy levels *in vivo*. Sections were then incubated with specific secondary horseradish peroxidase-conjugated antibodies (Dako, Carpinteria, CA, United States), photographed, and scored.

### Western Blot

Cells were treated with gefitinib, cycloheximide, MG132, and or HBSS for the indicated time period and then lysed using RIPA lysis buffer (Sangon Biotech) on ice. After the protein concentration was determined by BCA kit (Beyotime Technology), certain volume of protein was mixed with 4x Laemmli Sample Buffer (Bio-rad, CA, United States) for boiling water bath for 10 min for degeneration. The protein was separated by 10% sodium dodecyl sulfate-polyacrylamide gel electrophoresis and transferred onto polyvinylidene fluoride (PVDF) membranes (Millipore, Burlington, MA, United States). The PVDF membrane was blocked with 1% protein-free blocking buffer (Sangon Biotech). Strips of the appropriate size were cut out according to the marker and then incubated overnight at 4°C with specific antibodies, namely, LC3B, β-actin (all from Cell Signaling Technology), and P62 (Sigma, MO, United States). After washing TBST buffer, the PVDF strips were incubated with secondary horseradish peroxidase-conjugated antibodies for 2 h at room temperature. The protein bands were visualized using an electrochemiluminescence kit (Takara Bio, Shiga, and Japan), and their relative density was analyzed using ImageJ software. Autophagy related proteins were analyzed as introduced in the previous publication (Klionsky et al., 2021; Mizushima and Yoshimori, 2007).

For the immunoprecipitation assay, the GFP-LC3B plasmid was transfected into control cells and OE-CSP cells using a Lipofectamine 3000 kit (Thermo Fisher, Waltham, MA, United States). Six hour later, the cells were treated with MG132 or TAK-243 plus MG132 for 24 h and then collected, lysed, equalized, and incubated with GFP antibody (ratio 100:1, Invitrogen, CA, United States) for 2 h at 4°C. The protein was harvested and purified using a Capturem IP & Co-IP kit (Takara Bio) according for the manufacturer’s instructions. The protein was then separated by Western blotting and incubated with anti-LC3B or anti-ubiquitin antibody (Cell Signaling Technology, Beverly, MA, United States).

### Transcriptome Sequencing and Quantitative Reverse Transcription PCR (RT-qPCR)

A549 cells (1 × 10^6^) were seeded in 6-well plates and treated with HBSS for 24 h. The cells were lysed using TRIzol buffer, and transcriptome sequencing and analysis was performed by Oebiotech (Shanghai, China). Real-time quantitative reverse transcription polymerase chain reaction was performed to confirm the transcriptional level of ATG proteins. Briefly, the cells were treated with rapamycin, lysed with TRIzol buffer, and reverse-transcribed using a PrimeScript™ RT reagent kit with gDNA Eraser (Takara Bio). Real-time polymerase chain reaction (PCR) was then performed using a TB Green Fast qPCR kit (Takara Bio). The primers used in this study were as follows: *ATG3*, forward: 5′-GAC​CCC​GGT​CCT​CAA​GGA​A-3′, reverse: 5′-TGTAGCCC ATTGCCATGTTGG-3'; *ATG5*, forward: 5′-AAA​GAT​GTG​CTT​CGA​GAT​GTG​T-3′, reverse: 5′-CAC​TTT​GTC​AGT​TAC​CAA​CGT​CA-3'; *ATG7*, forward: 5′-ATG​ATC​CCT​GTA​ACT​TAG​CCC​A-3′, reverse: 5′-CAC​GGA​AGC​AAA​CAA​CTT CAAC-3'; *LC3B*, forward: 5′-GAT​GTC​CGA​CTT​ATT​CGA​GAG​C-3'; reverse: 5′-TTG​AGC​TGT​AAG​CGC​CTT​CTA-3'; *Beclin-1*, forward: 5′-CCA​TGC​AGG​TGA​GCT​TCG​T-3′, reverse: 5′-GAA​TCT​GCG​AGA​GAC​ACC​ATC-3'; *SQSTM1*, forward: 5′-GCA​CCC​CAA​TGT​GAT​CTG​C-3′, reverse: 5′-CGCTACACAAGTC GTAGTCTGG-3'.

### Public Database

Datasets (GSE38310, GSE75309, and GSE122005) were downloaded from the GEO database. Heatmap was created by R 4.03 with R package pHeatmap 1.0.12. Analyses of overall survival, progression-free survival, and post-progression survival in LUAD patients were performed with KMplot (KMplot.com).

### Statistical Analysis

All data are presented as the mean ± standard deviation. The proliferation, apoptosis, colony assay, and Western blotting experiments were repeated three times. The clones and relative grayscale values were analyzed using ImageJ software. The study data were analyzed and compared using SPSS software (version 19.0; IBM Corp., Armonk, NY, United States). Differences between groups were examined by one-way analysis of variance and unpaired two-tailed Student’s t-tests. Differences were considered statistically significant at *p* < 0.05.

## Results

### Overexpression of CSP Increased the Sensitivity of NSCLC Cells to Gefitinib *in vitro* and *in vivo*

**FIGURE 1:**
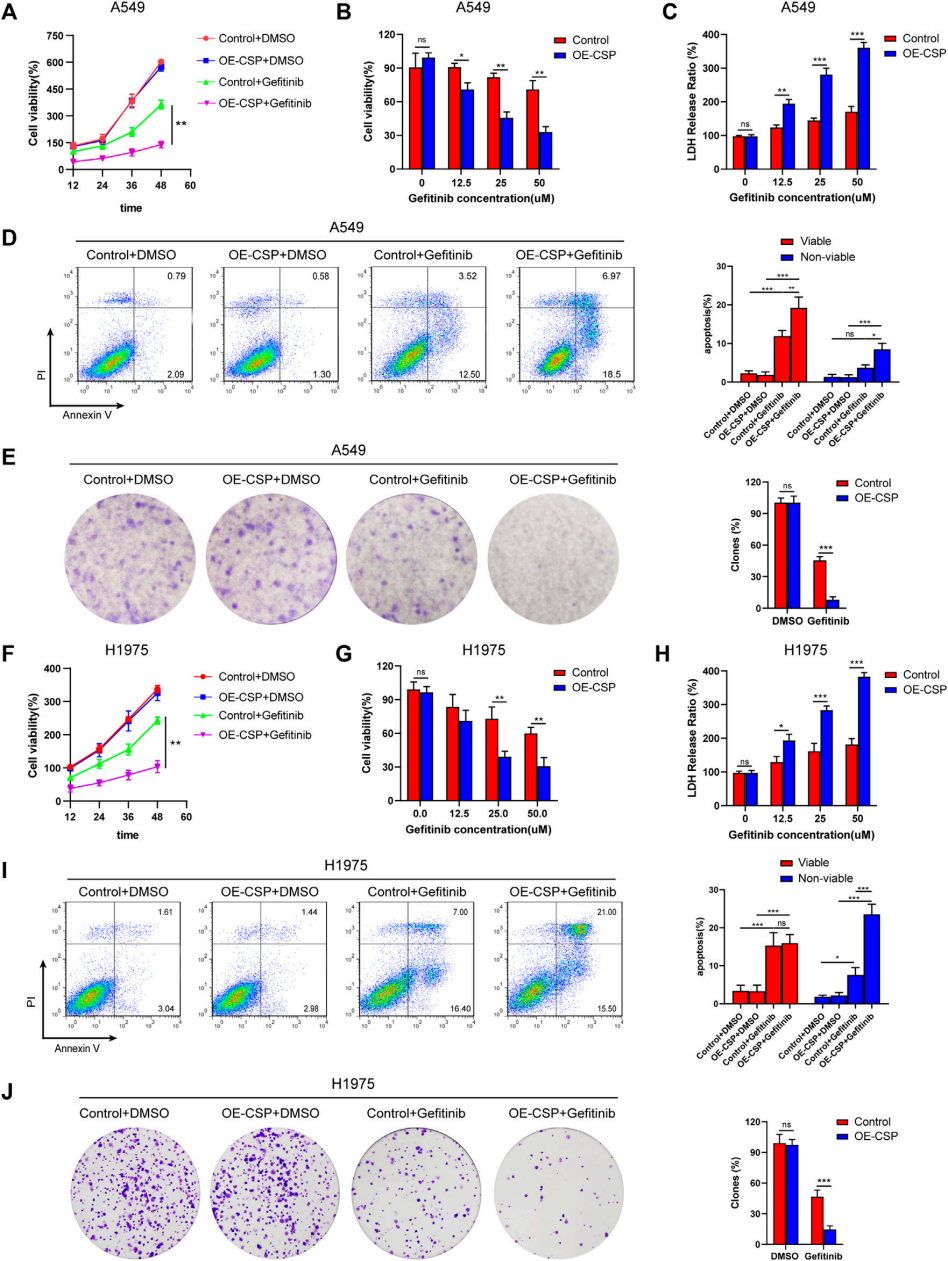
Overexpression of circumsporozoite protein (CSP) facilitated cytotoxicity caused by gefitinib. Control and OE-CSP A549 **(A)** and H1975 **(F)** cells (2 × 10^3^) were seeded in 96-well plates and treated with gefitinib. Cell viability was assessed at 12 h intervals. A549 **(B)** and H1975 **(G)** cells were treated with the indicated concentration of gefitinib for 24 h, and their viability was determined using a CCK8 kit and the lactate dehydrogenase assay **(C, H).** Next, control and OE-CSP A549 **(D)** and H1975 **(I)** cells (5 × 10^5^) were stained with Annexin V and propidium iodide after treatment with gefitinib. The apoptosis rate in the early and late stages was detected by flow cytometry. Representative images for flow cytometry (*left*) and statistical histograms (*right*). Viable, early apoptotic cells; non-viable, late apoptotic cells. Control and OE-CSP A549 **(E) **and H1975 **(J)** cells (1 × 10^3^) were then fixed in 4% paraformaldehyde and stained with 0.1% crystal violet solution. After incubation for 7 days, the colony number was analyzed using ImageJ software. The clone rate (%) was normalized to the control group (DMSO). Representative images for colony analysis (*left*) and statistical histograms (*right*). The data are presented as the mean ± standard deviation of three independent experiments *in vitro* (ns, not statistically significant, *p < 0.05, **p < 0.01, and ***p < 0.001).

To investigate the potential effect of CSP on sensitivity of NSCLC cells to gefitinib, we first constructed CSP stable expression A549 and H1975 cells. Exogenous CSP expression was verified by RT-qPCR and western blotting (Supplementary Figure S1). The anti-tumor activity of CSP was investigated by measuring changes in cell proliferation, apoptosis, and the ability of colony formation in A549 and H1975 (LUAD) cell lines when CSP was overexpressed. Cell viability was evaluated by CCK8 assay or LDH assay when cells were treated with various concentrations of gefitinib for 24 h or at different time points (Figures 1A–C, F–H). OE-CSP A549 and H1975 cells were more sensitive to gefitinib, and their viability was significantly lower than that of control cells. The survival rate of OE-CSP cells decreased in a concentration-dependent and time-dependent manner following gefitinib treatment. The findings for cell apoptosis and colony formation were consistent with those for cell viability. When cells were treated with gefitinib, the viable and non-viable apoptotic cell rates were significantly higher in the OE-CSP group (Figures 1D,I). The clone rate (%) was also lower in the OE-CSP group than in the control group (Figures 1E,J).

**FIGURE 2:**
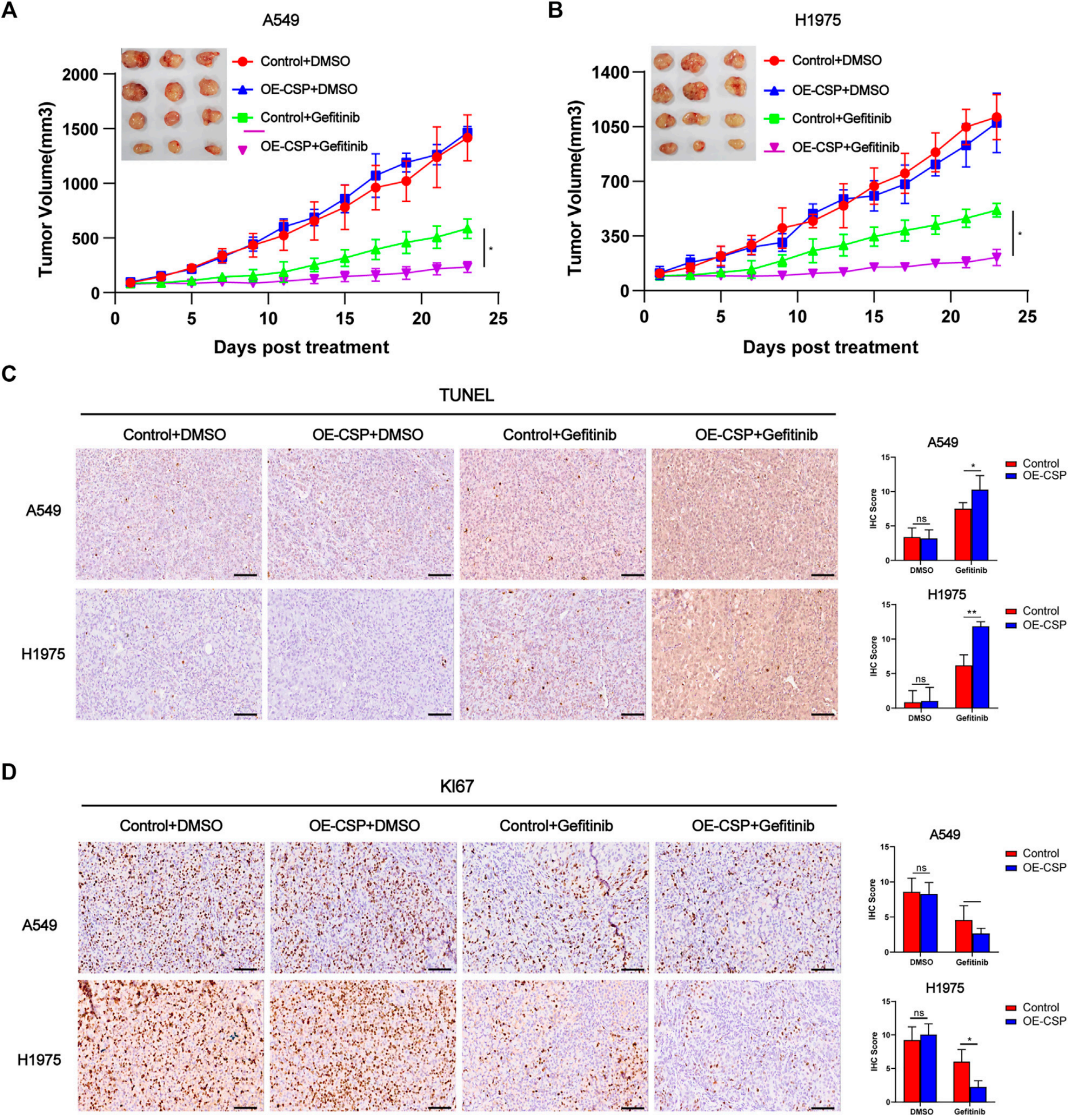
Overexpression of CSP enhances gefitinib-mediated inhibition of cell proliferation in vivo. Control and OE-CSP A549 **(A)** and H1975 **(B)** cells (5 × 10^6^) were subcutaneously injected into nude mice and gefitinib was started when the tumor volume approached 100 mm^3^. Experiments were repeated two times with three mice per group per experiment. The growth curves for the four groups were drawn and the final tumors were photographed. The tumor tissues were subjected to immunohistochemistry (IHC) staining for TUNEL **(C)** and Ki67 **(D)**. Representative images for IHC (*left*) and statistical histograms (*right*). Scale bar, 50 μm, n = 21 (*p < 0.05, **p < 0.01).

CSP-mediated enhance of gefitinib sensitivity was next confirmed in a subcutaneous tumor model. Nude mice were inoculated subcutaneously with control or OE-CSP cells and started on treatment with gefitinib on alternate days when the tumor volume reached approximately 100 mm^3^. After treatment with gefitinib, the growth of xenografts was significantly more suppressed, and the tumor volume was remarkably smaller in the OE-CSP group than in the control group (Figures 2A,B). Immunohistochemistry results of subcutaneous tumor showed that gefitinib caused pronounced cell death as a result of induction of cell apoptosis and inhibition of cell proliferation, with a greater amount of TUNEL (Figure 2C) and a lower Ki67 index in the OE-CSP group (Figure 2D). Together these findings indicate that CSP enhanced the sensitivity of LUAD cells to gefitinib and inhibited tumor cell growth both *in vivo* and *in vitro.*

### Circumsporozoite Protein Inhibited the Autophagy Induced by Gefitinib via Decreasing LC3B Protein

**FIGURE 3:**
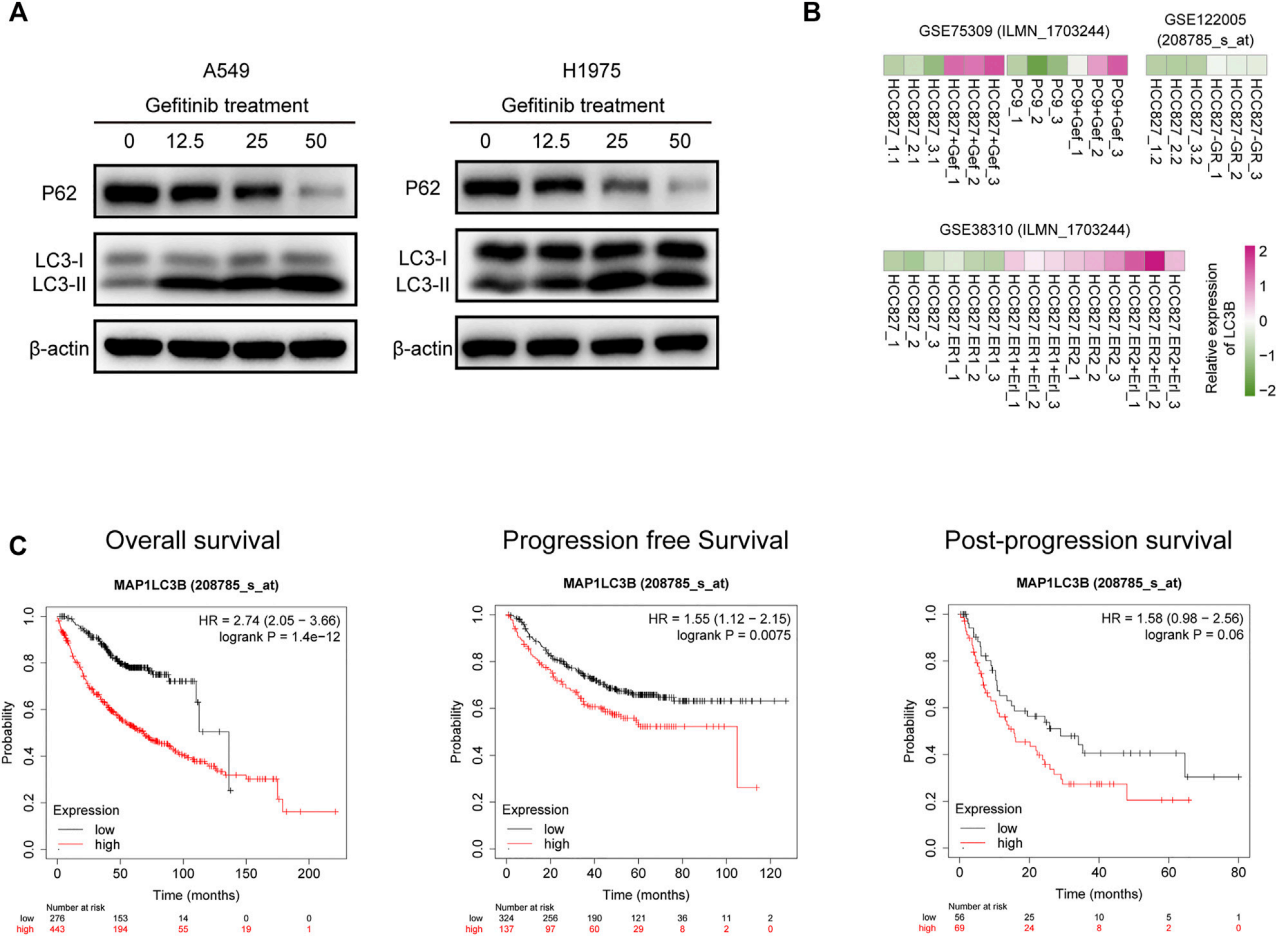
LC3B expression is upregulated by gefitinib treatment and negatively correlated with prognosis of LUAD patients. **(A)** A549 (*left*) and H1975 (*right*) cells were treated with the indicated concentrations of gefitinib for 24 h, LC3B and P62 protein level was detected by western blotting. **(B)** Heatmap represents LC3B mRNA expression in HCC827, HCC827-gefitinib resistance (HCC827-GR), and PC9 cells treated with or without gefitinib (Gef), and HCC827-erlotinib resistance cells (HCC827-ER) treated with or without erlotinib (Erl). **(C)** Correlation of LC3B expression and Overall survival (*left*), progression-free survival (*middle*), and post-progression survival (*right*) in LUAD patients.

Given that treatment with gefitinib contributed to induction of autophagy in several tumor models, we first assessed the effect of gefitinib on autophagy of A549 and H1975 cell. Western blotting analysis of LC3 and P62 confirmed that gefitinib treatment could induce autophagy activation in both A549 and H1975 cells in a dose-dependent manner (Figure 3A). Consistent with our result, data from three GEO datasets showed that LC3B expression was upregulated in HCC827-gefitinib resistance cells (HCC827-GR) and gefitinib treated HCC827 and PC9 cells, and the same phenomenon was seen in HCC827-erlotinib resistance cells (HCC827-ER) with erlotinib treated or not (Figure 3B). This result further confirmed autophagy level was enhanced in EGFR-TKIs treated or resistance LUAD cells. Moreover, patients with LC3B high expression had worse overall survival, progression-free survival, and post-progression survival than those with low expression of LC3B (Figure 3C), despite that the difference was not statistically significant in post-progression survival. These results indicate that gefitinib-induced autophagy is normally associated with its resistance in LUAD cells.

**FIGURE 4:**
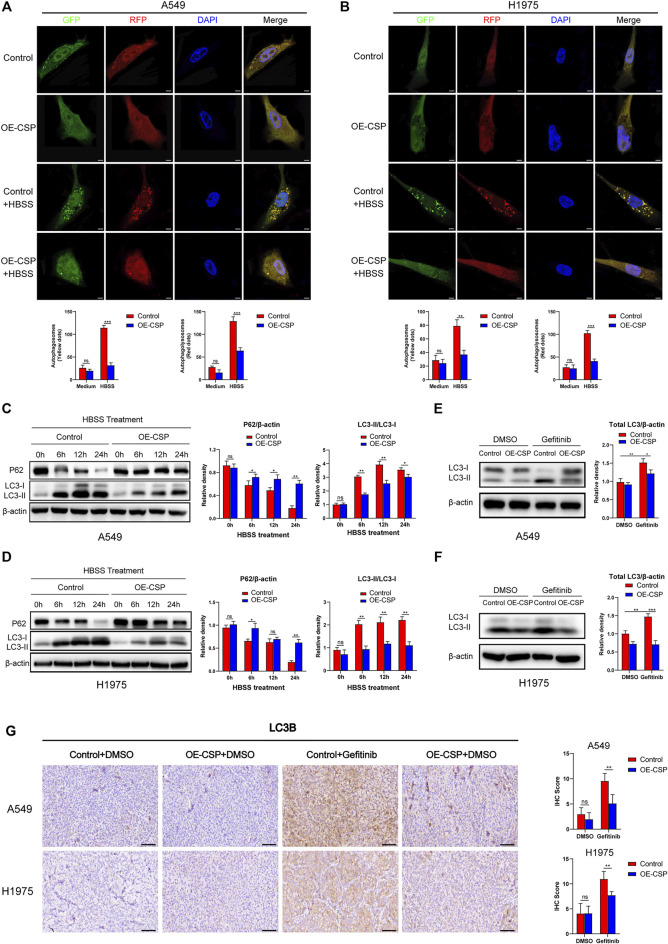
Overexpression of CSP inhibited cell autophagy both in vivo and *in vitro*. Control and OE-CSP A549 **(A)** and H1975 **(B)** cells (1 × 10^5^) were transfected with GFP/RFP-LC3B adenovirus and treated with HBSS for 24 h. Fluorescence was then detected using an immunofluorescence method. Representative images for immunofluorescence (*up*) and statistical histograms (*down*). The puncta of GFP-LC3B and RFP-LC3B were counted using ImageJ software. Scale bar, 5 μm. The protein level of LC3B and P62 in A549 **(C)** and H1975 **(D)** cells was detected using western blotting after they had been treated with HBSS for 0, 6, 12, and 24 h. The autophagy-related protein LC3B was detected after control and OE-CSP A549 **(E)** and H1975 **(F)** cells were treated with gefitinib. The relative density of LC3I/II, total LC3 and P62 was examined using ImageJ software. Representative images for western blotting (*left*) and statistical histograms (*right*). **(G)** Expression of LC3B protein in A549 and H1975 tumor tissues after treatment with gefitinib was measured by immunohistochemistry. Representative images for IHC (*left*) and statistical histograms (*right*). Scale bar, 50 μm, *n* = 21. Data are shown as the mean ± standard deviation of three independent experiments *in vitro* (ns, not statistically significant, *p < 0.05, **p < 0.01, and ***p < 0.001). HBSS, Hank’s balanced salt solution.

Then, we investigate whether CSP could inhibit autophagy in LUAD cells. When cells were infected with GFP/RFP-LC3B adenovirus and treated with HBSS for 24 h (a classical pathway for induction of autophagy), the number of both autophagosomes (yellow dots) and autophagolysosomes (red dots) was significantly decreased in OE-CSP group when cells were cultured with HBSS, which indicated the autophagy was inhibited after CSP overexpression (Figures 4A,B). Moreover, the decrease in LC3II/LC3I ratio and reduced degradation of p62 measured at different time points after treatment with HBSS also confirmed inhibition of autophagy in the OE-CSP LUAD cells (Figures 4C,D). We next compared LC3 levels in the OE-CSP and control groups after exposure to gefitinib. Although total LC3 was upregulated after treatment with gefitinib in control group, this increasing trend was significantly attenuated in the OE-CSP group (Figures 4E,F). Downregulation of LC3B protein in OE-CSP cells was also observed *in vivo*; subcutaneous tumors were isolated after gefitinib therapy, and immunohistochemical assays demonstrated that the immunohistochemistry score for LC3B protein was much lower in the OE-CSP group than in the control group (Figure 4G). These findings demonstrated that overexpression of CSP could suppress the autophagy induced by gefitinib through decreasing the LC3B protein.

### Circumsporozoite Protein Sensitized the Inhibitory Effect of Gefitinib by Suppressing Autophagy

**FIGURE 5:**
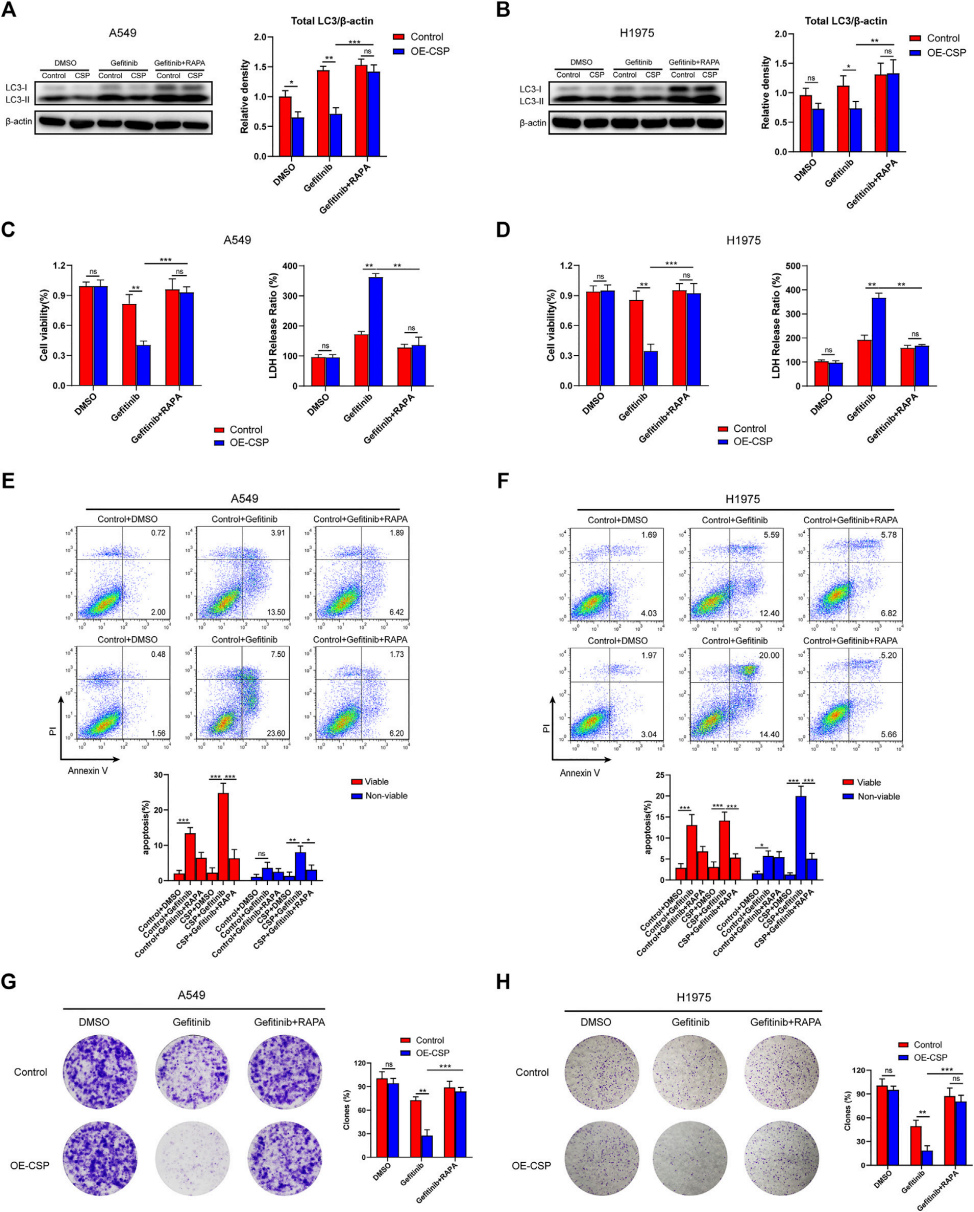
Inhibitory effect of gefitinib sensitized by CSP was mediated via suppression of autophagy. Control and OE-CSP A549 **(A)** and H1975 **(B)** cells (1 × 10^6^) were seeded in 6-well plates and treated with gefitinib alone or gefitinib plus rapamycin. The LC3B was detected by Western blotting and relative density of total LC3 was analyzed. Representative images for western blotting (*left*) and statistical histograms (*right*). The viability of **(C)** A549 and **(D)** H1975 cells was examined using a CCK8 kit (*left*) and LDH assay (*right*). For apoptosis, 5 × 10^5^ control and OE-CSP **(E)** A549 and **(F)** H1975 cells were treated with gefitinib alone or gefitinib plus rapamycin and stained with Annexin V and propidium iodide. The apoptosis rate was then detected using flow cytometry. Representative images for flow cytometry (*up*) and statistical histograms (*down*). Viable, early apoptotic cells; non-viable, late apoptotic cells. Colony formation assays were also performed for **(G)** A549 and **(H)** H1975 cells as above and the clones were counted using ImageJ software. The clone rate (%) was normalized to the control group (DMSO). Representative images for colony analysis (*left*) and statistical histograms (*right*). The data are presented as the mean ± standard deviation of three independent experiments *in vitro* (ns, not statistically significant, *p < 0.05, **p < 0.01, and ***p < 0.001).

To further confirm whether the ability of CSP to promote sensitivity to gefitinib was mediated by inhibiting autophagy, we treated cells with rapamycin, an inhibitor of the Akt/mTOR pathway, and to induce autophagy. As shown in Figures 5A,B, the total LC3B protein level was markedly increased in the OE-CSP group after treatment with rapamycin, indicating that rapamycin reverses the CSP-mediated downregulation of LC3B. As expected, although the sensitivity of the two OE-CSP LUAD cell strains to gefitinib increased markedly, there was no difference in cell viability (Figures 5C,D) or the rate of apoptosis (Figures 5E,F) between the control and OE-CSP groups after co-treatment with rapamycin and gefitinib. Moreover, the colony formation assay also did not reveal a statistically significant difference in the number of clones between the OE-CSP and the control group in response to treatment with gefitinib plus rapamycin, demonstrating that rapamycin treatment rescued the colony formed ability of OE-CSP cells (Figures 5G,H). These findings suggest that the sensitization to gefitinib in the OE-CSP group was mediated by repression of autophagy.

### Circumsporozoite Protein Enhanced Degradation of LC3B via the Ubiquitin-Proteasome Pathway

**FIGURE 6:**
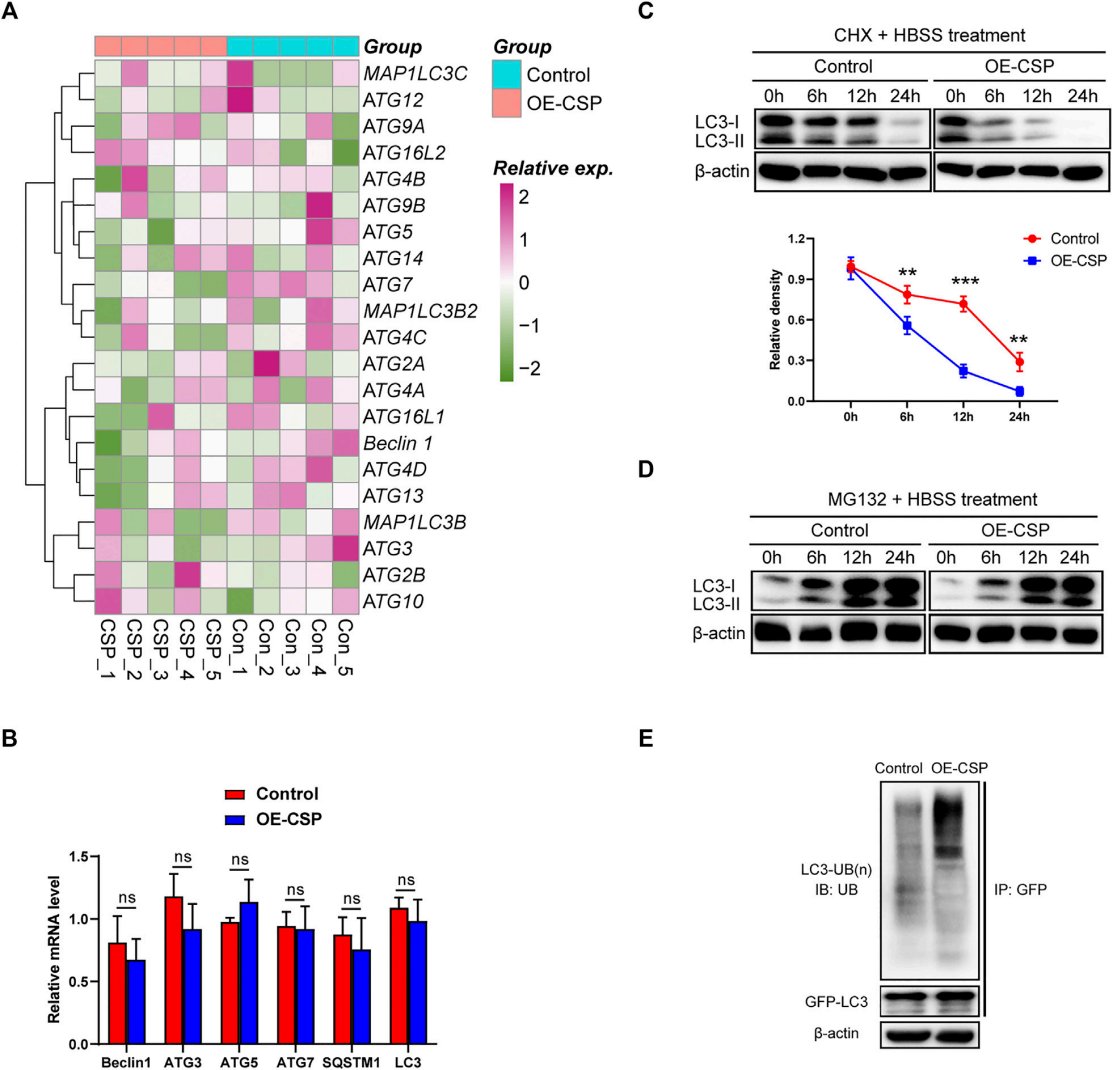
Overexpression of CSP promotes proteasomal degradation of LC3B. **(A)** Control and OE-CSP A549 cells (1 × 10^6^) were seeded in 6-well plates and treated with HBSS. They were then lysed with TRIzol buffer and transcriptome sequencing was performed. **(B)** The mRNA level of the autophagy-related proteins was confirmed using quantitative real-time polymerase chain reaction **(C)** Control and OE-CSP A549 cells were treated with cycloheximide and the protein was collected 0, 6, 12, and 24 h later. The LC3B protein level (*up*) was then detected and the half-life period (*down*) was analyzed. **(D)** After treatment with MG132 for 0, 6, 12, and 24 h, the LC3B protein level was detected using Western blotting. **(E)** Control and OE-CSP A549 cells (1 × 10^6^) were transfected with GFP-LC3B plasmid; they were then lysed with Western lysis buffer and the GFP-LC3B protein was purified using a Capturem IP & Co-IP kit. The ubiquitin level was detected. The data are presented as the mean ± standard deviation of three independent experiments *in vitro* (ns, not statistically significant, *p < 0.05, **p < 0.01, and ***p < 0.001).

The mRNA levels of LC3 and other autophagy-related proteins from the OE-CSP and control A549 cells were sequenced and compared to explore the underlying mechanism of CSP-mediated autophagy suppression. As shown in Figure 6A, overexpression of CSP had no significant effect on ATG gene mRNA levels overall. A subsequent real-time PCR experiment further confirmed this result (Figure 6B), suggesting that the decrease in LC3B protein in OE-CSP A549 cells was not mediated at the transcription stage. The ubiquitin-proteasome and autophagy-lysosome pathways are the two main routes of protein degradation in eukaryotes (Varshavsky, 2017). Interestingly, autophagy was inhibited in OE-CSP LUAD cells, and suggesting that the autophagy-lysosome pathway is not involved in reduction of the LC3B protein level. Therefore, we examined whether the decreased expression of LC3B was induced by the ubiquitin-proteasome pathway. The half-life of LC3B was detected by western blotting at different time points after the cells were treated with cycloheximide (CHX). We found that the half-life of LC3B was significantly shorter in the OE-CSP group than in the control group after treatment with HBSS (Figure 6C), and that the protein was aggregated when cells were treated with the proteasome inhibitor MG132 (Figure 6D). Moreover, the ubiquitination level of LC3B in the OE-CSP A549 cells was much higher than that in the control cells (Figure 6E). Our data suggest that degradation of LC3B protein by CSP may be mediated by the ubiquitin-proteasome pathway.

### Ubiquitin Inhibition by TAK-243 Rescues the Sensitizing Effect of Circumsporozoite Protein on Gefitinib

**FIGURE 7:**
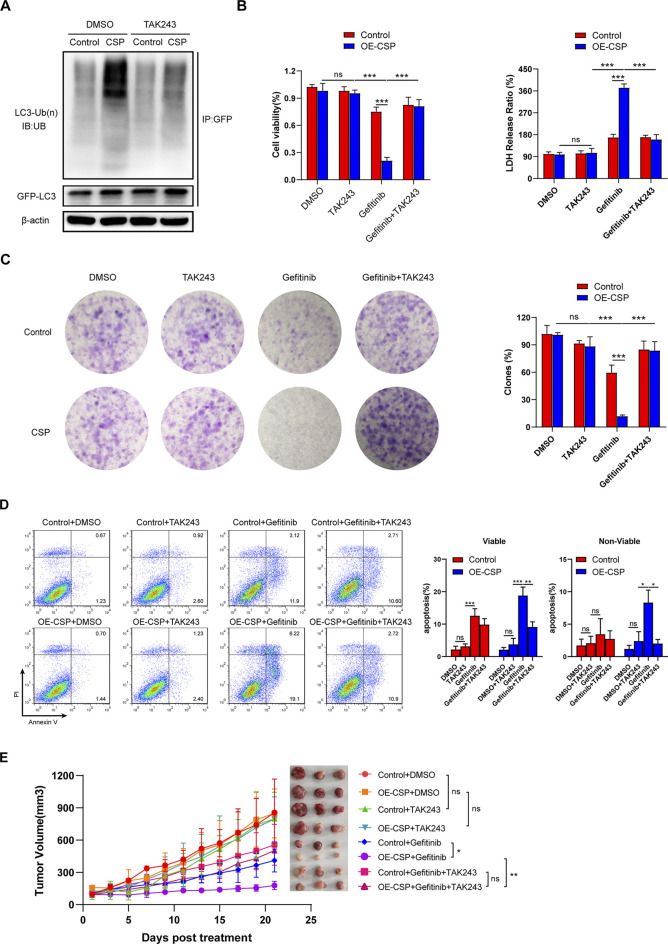
Ubiquitin inhibition by TAK-243 rescues the sensitizing effect of CSP on gefitinib. **(A)** Control and OE-CSP A549 cells (1 × 10^6^) were transfected with GFP-LC3B plasmid and then treated with TAK-243 and MG132 for 24 h. The GFP-LC3B was purified and the level of ubiquitin was detected by Western blotting. Cell viability after treatment with TAK-243 and gefitinib was examined by **(B)** a CCK8 kit (*left*) and LDH assay (*right*). **(C)** Cell colony formation ability and **(D)** apoptosis were also assessed using the method described above. The clone rate (%) was normalized to the control group (DMSO). Representative images for colony analysis and flow cytometry (*left*) and statistical histograms (*right*). Viable, early apoptotic cells; non-viable, late apoptotic cells. **(E)** Nude mice were then treated with TAK-243 plus gefitinib when the tumor volume was approaching 100 mm^3^. Experiments were repeated two times with three mice per group per experiment. The data are presented as the mean ± standard deviation of three independent experiments *in vitro* (ns, not statistically significant, **p < 0.01, ***p < 0.001).

To further confirm that CSP overexpression can sensitize LUAD cells to inhibition by gefitinib via enhancing ubiquitin degradation of LC3B, we examined cell viability, apoptosis, and the ability of colony formation by inhibiting the cell ubiquitin-activating enzyme using TAK-243, a small-molecule inhibitor that disrupts all ubiquitin signaling, and global protein ubiquitination (Hyer et al., 2018). Given the cytotoxicity of TAK-243 in tumor cells, we first treated A549 cells with various concentrations of TAK-243 to detect cell viability and selected a concentration of 1 µM because it did not interfere with cell survival (Supplementary Figure S2). As shown in Figure 7A, the ubiquitylation level of LC3B decreased significantly in OE-CSP A549cells after they were treated with TAK-243. The viability (Figure 7B) and colony formation ratio (Figure 7C) of OE-CSP A549 cells were comparable with those of control cells after treatment with gefitinib plus TAK-243, as was the cell apoptosis rate (Figure 7D). To further confirm the role of ubiquitylation in the response of CSP-sensitized lung cancer cells to gefitinib *in vivo*, we intravenous injected TAK-243 twice a week during treatment with gefitinib. Injection of TAK-243 significantly inhibited the anti-tumor efficiency of gefitinib in the OE-CSP group, and the tumor volume was larger than that in the gefitinib group (Figure 7E).

## Discussion

Clinical trials using autophagy-targeting drugs either as monotherapy or in combination therapy with EGFR-TKIs are undergoing. Although some studies have shown these autophagy inhibitors to have promising effects in patients with intractable disease, side effects such as retinopathy and gastrointestinal problems due to agents such as hydroxychloroquine and chloroquine are an issue, and the appropriate indications for these drugs are not yet determined (Deng et al., 2019; Mulcahy and Thorburn, 2020; Uberall et al., 2019). An increasing number of studies have found that attenuated parasites (Zhou et al., 2019), blood-stage malarial parasites (Liu et al., 2017), anti-parasitic agents (Li et al., 2021; Lin et al., 2016; Vatsveen et al., 2018), and parasite proteins (Lopez et al., 2010; Ramirez-Toloza et al., 2020) had anti-tumor activity in various tumor models. These reports point to the possibility of identifying anti-tumor strategies from the perspective of parasites. In this study, we found that overexpression of *Plasmodium* CSP in the cytoplasm of LUAD cells could suppress the autophagy induced by gefitinib and play an active role in enhancing apoptosis and suppressing proliferation and colony formed ability of LUAD cells via synergistic interaction. Interestingly, this phenomenon was observed in both EGFR-mutant H1975 cell and EGFR-wildtype/KRAS-mutant A549 cell, indicating CSP sensitization to EGFR-TKIs might applies to different EGFR gene mutation status. Moreover, we noticed that overexpression of CSP did not induce any cytotoxicity in LUAD cells either *in vitro* or *in vivo*. This might be related to its ability to protect cells from death caused by invasion of sporozoites (Zheng et al., 2014). Therefore, suppression of CSP-mediated autophagy and low cell toxicity indicate that it is a promising sensitizer for EGFR-TKIs.

LC3B is a structural protein in autophagosomal membrane and exerts a crucial role during autophagy progress. Upregulation of LC3B can be found in gefitinib or erlotinib treated and resistance LUAD cells, meanwhile, high LC3B level also is associated with worse progression-free and post-progression survival in LUAD patients (Figure 3). These results indicated that downregulation of LC3 protein might be a potential strategy to improve efficacy of EGFR-TKIs. Autophagy and proteasomes are the most common pathways for degradation of protein and cooperate to maintain cell and protein homeostasis (Dikic, 2017). However, the difference is that the ubiquitin-proteasome system mainly degrades soluble monomers and misfolded proteins, whereas autophagy predominantly degrades protein aggregates and damaged organelles (Athane et al., 2015; Wang and Mandelkow, 2012). In the past, autophagy and the proteasome were deemed to be independent of each other, whereas accumulating evidence now shows crosstalk between these degradation pathways (Cohen-Kaplan et al., 2016), including sharing and co-degradation of substrates (Scotter et al., 2014; Song et al., 2016; Watanabe et al., 2016; Yang et al., 2013). In this study, we demonstrated LC3B was downregulated by CSP via the ubiquitin-proteasome system but could not identify the mechanisms regulating LC3B ubiquitination in OE-CSP LUAD cells. LC3B is usually degraded in autophagolysosomes during the process of autophagy; however, cellular autophagy was inhibited in OE-CSP LUAD cells in our study, suggesting degradation of autophagy is not responsible for the decreased amount of LC3B protein. Recent studies also provided evidence that the LC3B protein level in cytoplasm was also controlled by ubiquitin enzyme and deubiquitinase (Jia and Bonifacino, 2019; Jia and Bonifacino, 2021). Therefore, more research is needed to determine whether or not CSP can regulate the action of these enzymes.

Bioimmunotherapy is another novel treatment for cancer that has a significant curative effect by activating the autoimmune response against tumor cells (Rosenberg, 2005; Tsao et al., 2016). Use of parasites as antigens to induce the host immune response and exert an anti-tumor effect is now a promising area of research. Experiments have shown that attenuated *Plasmodium* sporozoites and *Toxoplasma gondii* or Toxoplasma antigen have an inhibitory effect in lung cancer (Zhou et al., 2019), melanoma (Baird et al., 2013a), pancreatic cancer (Sanders et al., 2015,^2016^), ovarian cancer (Baird et al., 2013b), and colon cancer (Kim et al., 2020). CSP has become a prime vaccine candidate for malaria based on its importance in the physiological processes of the sporozoite. Previous studies have confirmed that the abundant epitopes of CSP trigger the immune response (Agnandji et al., 2011) and induce robust CD8^+^ T-cell responses that are capable of eliminating developing parasites in hepatocytes, resulting in protective immunity (Gibbins et al., 2020). In addition, cancer gene therapy also provide an approach, including immune gene therapy, and to suppress tumor growth with other conventional therapy (Cemazar et al., 2017). Technically, CSP can be delivered into tumor cells by nanomaterials packaged with CSP mRNA and viral vectors carrying CSP protein, which sheds us new therapeutic strategies to overcome EGFR-TKI resistance in advanced NSCLC patient with EGFR mutant. Furthermore, it has been reported previously that autophagy inhibition in lung cancer can switch tumor associated macrophages to express the M1 phenotype, thus enhancing antitumor T cell-mediated immunity of anti-PD1 therapy (Kuo et al., 2021; Li et al., 2018). The findings of these studies have led to the interesting hypothesis that overexpression of CSP in lung cancer cells could induced a CSP peptide-specific CD8^+^ T-cell response that combats tumor growth. Unfortunately, this hypothesis could not be tested in this study because immunodeficient mice were used but will be explored in the future.

In this study, we found that CSP enhanced drug sensitivity and inhibited the autophagy induced by gefitinib in NSCLC cells and was mediated by the ubiquitin-proteasome system.

## Data Availability

The datasets presented in this study can be found in online repositories. The names of the repository/repositories and accession number(s) can be found below: https://www.ncbi.nlm.nih.gov/geo/, GSE189311.
