# Supplementary material for: Plasmodium Circumsporozoite Protein Enhances the Efficacy of Gefitinib in Lung Adenocarcinoma Cells by Inhibiting Autophagy via Proteasomal Degradation of LC3B
Source: Front Cell Dev Biol. 2022 Feb 3;10:830046. doi: 10.3389/fcell.2022.830046 (PMC8851824; doi:10.3389/fcell.2022.830046)
Supplement: Supplementary file 1 [file DataSheet1.PDF]

Supplementary Material

Supplementary Figures

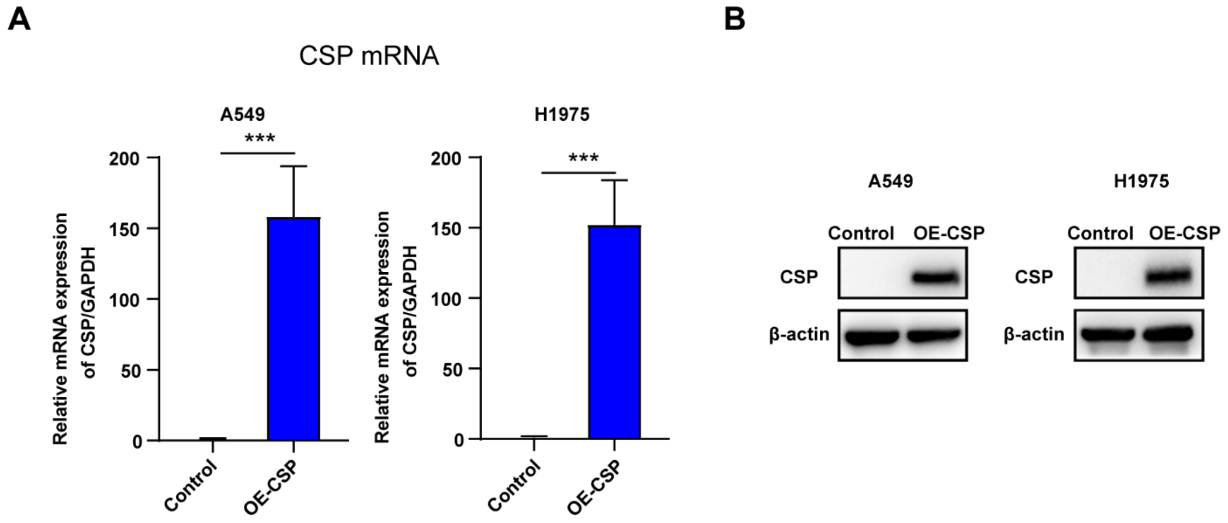

Figure S1 Exogenous CSP expression in CSP stable expression LUAD cells.

Control and CSP stable expression A549 and H1975 cells (1×10^6) were collected to verify the expression of CSP. The mRNA and protein level of CSP was measured by qPCR (**A**) and western blotting (**B**) in CSP stable expression A549 and H1975 cells. Primers for CSP, Fw:5’-ACAACAGCCACCACAACAAC-3'; Rv: 5’-CACTACATTGAGACCATTCCTCTG-3'. GAPDH was used as the internal reference. Primary polyclonal antibody for CSP (anti-CSP-repeat-region) was a gift from Prof. Wenyue Xu (Army Medical University). ***P<0.001.

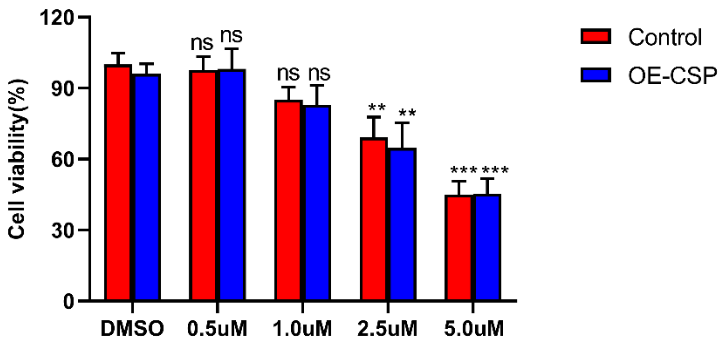

Figure S2 Cytotoxic effect of TAK-243 at the indicated concentrations.

Cells (2×10^3) were seeded in 96-well plates and cultured for 12 h. For detection of viability, the cells were treated with various concentrations of TAK-243 for 24 h for the indicated time points. Cell viability was examined using a CCK8 kit (Beyotime Biotechnology, Jiangsu, China). Two-three independent experiments were performed for each experiment. Data are represented as mean ± SEM, and analyzed by One-way ANOVA. Each group was compared to corresponding DMSO group; ns, not significant; *$P < 0.05$; **$P<0.01$; ***$P<0.001$.
